# Jugular venous reflux and plasma endothelin-1 are associated with cough syncope: a case control pilot study

**DOI:** 10.1186/1471-2377-13-9

**Published:** 2013-01-16

**Authors:** Chih-Ping Chung, Chun-Yu Cheng, Robert Zivadinov, Wei-Chih Chen, Wen-Yung Sheng, Yu-Chin Lee, Han-Hwa Hu, Hung-Yi Hsu, Kuang-Yao Yang

**Affiliations:** 1 Department of Neurology, Taipei Veterans General Hospital, Taipei, Taiwan; 2 School of Medicine, National Yang Ming University, Taipei, Taiwan; 3 Buffalo Neuroimaging Analysis Center, and the Department of Neurology, State University of New York at Buffalo, New York, USA; 4 Department of Respiratory Therapy, Taipei Veterans General Hospital, Taipei, Taiwan; 5 Division of Pulmonary Medicine, Department of Medical Affairs, Taipei Municipal Gan-Dau Hospital, Taipei, Taiwan; 6 Department of Chest Medicine, Taipei Veterans General Hospital, Taipei, 112, Taiwan; 7 Immunology Center, Taipei Veterans General Hospital, Taipei, Taiwan; 8 Institute of Clinical Medicine, Infection and Immunity Research Center, School of Medicine, National Yang Ming University, Taipei, Taiwan; 9 Department of Neurology, Tungs' Taichung Metro Harbor Hospital, Wuci Township, Taichung County, Taiwan; 10 School of Medicine, Chung Shan Medical University, Taichung, Taiwan; 11 Department of Neurology, Taichung Veterans General Hospital, Taichung, Taiwan

**Keywords:** Cough syncope, Endothelin-1, Jugular venous reflux

## Abstract

**Background:**

Jugular venous reflux (JVR) has been reported to cause cough syncope via retrograde-transmitted venous hypertension and consequently decreased cerebral blood flow (CBF). Unmatched frequencies of JVR and cough syncope led us to postulate that there should be additional factors combined with JVR to exaggerate CBF decrement during cough, leading to syncope. The present pilot study tested the hypothesis that JVR, in addition to an increased level of plasma endothelin-1 (ET-1), a potent vasoconstrictor, is involved in the pathophysiology of cough syncope.

**Methods:**

Seventeen patients with cough syncope or pre-syncope (Mean[SD] = 74.63(12.37) years; 15 males) and 51 age/gender-matched controls received color-coded duplex ultrasonography for JVR determination and plasma ET-1 level measurements.

**Results:**

Multivariate logistic analysis showed that the presence of both-side JVR (odds ratio [OR] = 10.77, 95% confident interval [CI] = 2.40-48.35, *p* = 0.0019) and plasma ET-1 > 3.43 pg/ml (OR = 14.57, 95% CI = 2.95-71.59, *p* = 0.001) were independently associated with the presence of cough syncope/ pre-syncope respectively. There was less incidence of cough syncope/ pre-syncope in subjects with the absence of both-side JVR and a plasma ET-1 ≦3.43 pg/ml. Presence of both side JVR and plasma ET-1 level of > 3.43 pg/ml, increased risk for cough syncope/pre-syncope (*p* < 0.001).

**Conclusions:**

JVR and higher plasma levels of ET-1 are associated with cough syncope/ pre-syncope. Although sample size of this study was small, we showed a synergistic effect between JVR and plasma ET-1 levels on the occurrence of cough syncope/pre-syncope. Future studies should confirm our pilot findings.

## Background

Syncope is defined as a transient loss of consciousness with subsequent spontaneous recovery, resulting from global cerebral hypoperfusion [1]. Besides syncope, a lesser degree of compromised cerebral blood flow (CBF) may lead to pre-syncope symptoms, such as wooziness, light-headedness, nearly fainting sensation and/or visual dimming. Cough syncope refers to syncope upon coughing, [2] the pathogenesis of which is unclear and probably multifactorial.

Endothelin-1 (ET-1) is a potent vasoconstrictor peptide derived from vascular endothelial cells [3]. Besides its direct vasoconstriction effect, increased ET-1 levels may result in decreased nitric oxide (NO) availability, thereby predisposing to vasoconstriction [4,5]. ET-1 also contributes to the regulation of cerebral vascular tone. ET-1 levels have been shown as elevated in the plasma and cerebrospinal fluid (CSF) of subarachnoid hemorrhage (SAH) patients, with the presence of elevated ET-1 levels correlating with the persistence of cerebral vasospasm [4,6,7]. Additionally, ET-1 levels have been observed to decline in the absence of cerebral vasospasm, [7] and the administration of ET-1 antagonists prevents cerebral vasospasm [8]. Therefore, ET-1 is one of determinants of CBF. Endothelial dysfunction with imbalanced releases of NO and ET-1 has also been recognized in chronic obstructive pulmonary disease (COPD); those populations have been found with more frequent cough syncope [9,10].

Jugular venous reflux (JVR) is found frequently in transient global amnesia, [11] transient monocular blindness patients, [12] and elderly people with more severe age-related white matter changes [12]. JVR may occur during a Valsalva maneuver (VM) or Valsalva-like activities, such as cough, when increased intrathoracic pressure is beyond the competence of internal jugular venous (IJV) valves. VM-induced JVR, which may retrogradely transmit venous hypertension into cerebral venous system, decreases cerebral perfusion pressure (CPP) and consequently reduces CBF during Valsalva-like activities [13-16]. It has been suggested that JVR plays a role in the pathophysiology of cough syncope [17]. However, the low incidence of cough syncope, disproportionate to the higher frequency of JVR, [18] implies that additional factors should be involved. In the present study, we hypothesized that (1) the presence of JVR is associated with cough syncope, and (2) there is an interactive effect between JVR and the plasma ET-1 levels on the occurrence of cough syncope.

## Methods

### Subjects

This was a prospective case–control pilot study. For valid statistical analyses, the numbers of recruited cough syncope/pre-syncope cases (case group) and age/gender-matched controls (control group) were set as 1:3. Between July/2009 and August/2010, Taiwanese patients consecutively enrolled at the Neurological and Chest Outpatient Clinics of Taipei Veterans General Hospital with cough syncope/pre-syncope were assessed for inclusion into this study. The definition of cough syncope and cough pre-syncope were defined as the presence of consciousness loss, and the presence of wooziness, light-headedness, a nearly fainting sensation and/or visual dimming without consciousness loss, respectively, during involuntary coughing. Arterial blood pressure (ABP) measurement, eletrocardiography (EKG), eletroencephalography (EEG) and neck vascular duplex sonography were performed in all patients. Subjects eligible for participation in the current study had normal EKG and EEG studies, and the absence of neck arterial stenosis by duplex sonography. Exclusion criteria (including the control group) were a past history of stroke, ischemic heart disease, congestive heart disease, valvular heart disease, cardiac arrhythmia, or malignancy. Upon recruiting one eligible cough syncope/pre-syncope case, three age/gender-matched controls were recruited from patients who visited the Chest Outpatient Clinic due to cough but no cough-related neurological symptoms. The study protocol was approved by the Institutional Review Board of Taipei Veterans General Hospital and was conducted in accordance with the Helsinki Declaration. Written informed consent was obtained from all participants or their authorized representatives before enrollment.

Vascular risk factors were defined according to international guidelines [19]. COPD was diagnosed per the definitions in the Global Initiative for Chronic Obstructive Lung Disease guidelines [20].

### JVR determination

**Figure 1:**
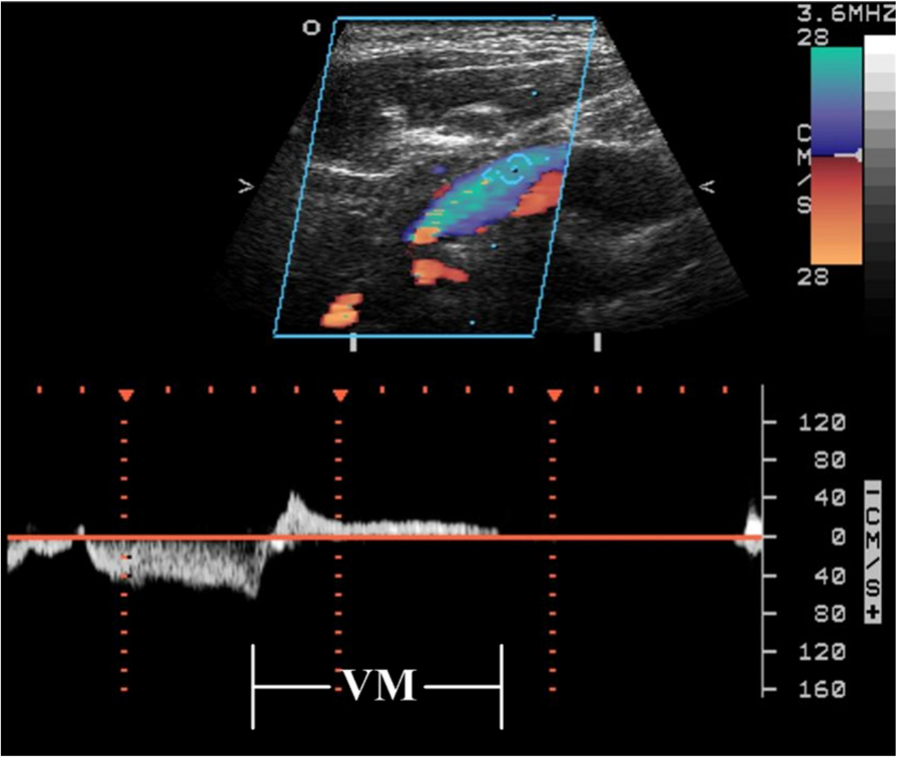
Retrograde flow detected by color duplex and in the Doppler spectrum during Valsalva maneuver (VM) is considered as jugular venous reflux (JVR).

Neck color-coded duplex sonography was performed in all subjects with a 7-MHz linear transducer (Sonos 5500, Hewlett Packard, Andover, MA, USA) by the same technician, who was blinded to subjects’ characteristics. The examination was done at least 2 hours after a light breakfast in the morning. On examination, subjects were in a head-straight, flat supine position after a quiet 10 minute rest. The IJV was initially insonated longitudinally and thoroughly from the proximal part of the neck base rostrally to the distal part at the submandibular level in order to detect any possible spontaneous JVR at baseline [11]. Then, the VM was performed by forcible expiration from subject’s mouth into a flexible rubber tube connected to a manometer. Subjects were asked to reach 40 mm Hg Valsalva pressure and maintain it for at least 10 seconds. During the VM, the distal margin of the window of the color signal was placed at the tip of the flow divider of the internal carotid artery. The color box was adjusted to include the entire lumen of the IJV; if retrograde color appeared in the center of the lumen, the retrograde flow would then be confirmed by Doppler spectrum. JVR was determined when the retrograde-flow color in the center of the lumen and the Doppler-flow waveform demonstrated reversal of flow for more than 0.5 seconds at baseline and/or during the VM (Figure 1) [11,15,21,22].

### Plasma ET-1 levels measurement

Non-fasting venous blood samples were drawn before neck color-coded duplex sonography in all subjects, at a similar morning time to avoid a circadian variation and collected in the tubes containing EDTA. Plasma was separated from whole blood and stored at −80°C until analysis. Plasma levels of ET-1 were measured using a commercially available immunoassay kit (R&D Systems Inc., Minneapolis, MN, USA). The intra- and interassay coefficients of variation of ET-1 in our laboratory were 4.4% and 5.7%. All measurements were performed in duplicate.

### ABP changes during VM

Since VM/cough-induced hypotension is presumed as one possible mechanism in cough syncope, [23,24] we also recorded ABP changes during the VM, a maneuver that mimics the effect of cough, by servocontrolled infrared finger plethysmography (Finapres, model 2300, Ohmeda Monitoring Systems, Englewood, CT, USA.) in patients with cough syncope and age/gender-matched controls. Experiments were performed in the morning at least 2 hours after a light breakfast. After at least 10 minutes of supine rest, two VMs were performed with a 5-minute interval between them. The second VM was used for data analysis. The VM was performed for a duration of 15 seconds, with intrathoracic pressure of 40 mmHg maintained and monitored by a pressure gauge connected to a flexible tube.

Beat-to-beat ABP measured for 15 seconds before the VM was averaged as baseline. Phasic changes in mean ABP with the VM were defined previously [15,25]. There are four distinct well described phases of ABP changes. With the beginning of the strain (phase I), there is a transient increase in ABP resulting from transmission of intrathoracic pressure to the arterial system. At phase II, continuously increased intrathoracic pressure will impede venous return to the heart and lead to a fall in ABP early in phase II (phase IIa). Then in late phase II (phase IIb), a sympathetic response to the fall in ABP will produce a rise in ABP. When the strain is relieved (phase III), more venous blood volume pools in the expanded intrathoracic veins due to sudden intrathoracic pressure decrement, which decreases left atrial filling and, subsequently, ABP. This is followed by an overshoot in ABP (phase IV), when atrial filling is normalized accompanied by remain-elevated sympathetic tone. Relative changes in mean ABP at each phase of the VM are calculated as the ratio of the magnitude of the phasic changes at each phase divided by the baseline measurements.

### Statistical analysis

Continuous data are expressed as mean (SD). The nonparametric Mann–Whitney *U* test was used to compare the case group and the control group. The x^2^ test was used for evaluating categorical variables, and Fisher’s exact test was used for instances in which individual counts in any group were fewer than five. Univariate and multivariate logistic analyses were performed using odds ratio (OR) with a 95% confidence interval (CI) to test the independent effect of factors associated with cough syncope/pre-syncope. To determine the predictive accuracy of ET-1 level for cough syncope, receiver operating characteristic (ROC) curve was constructed, and the area under the curve (AUC) was calculated. Based on the optimal cut-off value of plasma ET-1 level determined from the ROC curve, in univariate and multivariate analyses, we took 3.43 pg/ml as the cut-off point. Among JVRs of different severities and sides, we used the presence of both-side JVR to run the univariate and multivariate analyses. To test the interactive effects between JVR and the levels of plasma ET-1 on the presence of cough syncope/pre-syncope, we divided all subjects into four groups. Those groups were (1) the presence of both-side JVR with plasma ET-1 > 3.43 pg/ml; (2) the absence of both-side JVR with plasma ET-1 > 3.43 pg/ml; (3) the presence of both-side JVR with plasma ET-1 ≦3.43 pg/ml; and (4) the absence of both-side JVR with plasma ET-1 ≦3.43 pg/ml. Then we used Mantel-Haenszel x^2^ to test for trends across these four groups in the frequency of cough syncope/pre-syncope. For all tests, *p* < 0.05 was considered statistically significant. All analyses were performed with SAS software, version 9.1 (SAS Institute Inc, Cary, NC).

## Results

### Patient characteristics

All cough syncope/pre-syncope cases presented with first-time, cough-related neurological symptoms. We excluded one patient with severe internal carotid artery stenosis, and another patient with seizure history. In the end, there were 10 patients with cough syncope, 7 patients with cough pre-syncope, and 51 age/gender-matched control subjects recruited in the present study. In subjects with cough pre-syncope, upon cough all had a nearly fainting sensation (light-headedness, dizziness, etc.) and five (71.43%) had visual dimming. All subjects in the case group developed a neurological symptom during a prolonged or repetitive vigorous cough. They did not experience syncope/pre-syncope at other times. Subjects with cough syncope/pre-syncope also had no vertigo, tinnitus, and evidence of convulsion or incontinence during cough. The demographic/clinical characteristics are demonstrated in Table 1. The severity of COPD was similar between case and control groups. There were no significant differences in the demographic factors between case and control groups. In addition, there were no differences in the demographic characteristics, the frequencies of JVR, and plasma ET-1 levels between subjects with cough syncope and cough pre-syncope (Mann–Whitney *U* and x^2^ test; data not shown).

**Table 1:** The characteristics and the frequencies of jugular venous reflux and plasma endothelin-1 levels in cough syncope/pre-syncope patients and control subjects

	**Cough syncope/pre-syncope (n = 17)**	**Controls (n = 51)**	**p value**
Age, mean (SD), yr	74.63 (12.37)	74.63 (12.37)	
Gender, M/F	15/2	45/6	
Vascular risk factors, n (%)			
HTN	4 (23.53%)	10 (19.61%)	0.729
Diabetes mellitus	1 (5.88%)	5 (9.80%)	0.365
Hyperlipidemia	0	4 (7.84%)	0.307
Smoking	13 (76.47%)	41 (80.39%)	0.729
COPD	14 (82.35%)	34 (66.67%)	0.219
GOLD I	6 (43%)	15 (44%)	
GOLD II	6 (43%)	12 (35%)	
GOLD III	2 (14%)	7 (21%)	
Right-side JVR, n (%)	13 (76.47%)	25 (49.02%)	0.048
Left-side JVR, n (%)	11 (64.71%)	18 (35.29%)	0.034
Both-side JVR, n (%)	10 (58.82%)	8 (15.69%)	0.0005
Plasma ET-1 level, pg/ml	3.64 (1.23)	2.39 (1.01)	<0.0001

Abbreviations: HTN = hypertension; COPD = chronic obstructive pulmonary disease; GOLD = Global Initiative for Chronic Obstructive Lung Disease guideline; JVR = jugular venous reflux; ET-1 = endothelin-1; SAH, subarachnoid hemorrhage.

### JVR and plasma ET-1 level comparisons between cough syncope/pre-syncope and control groups

**Figure 2:**
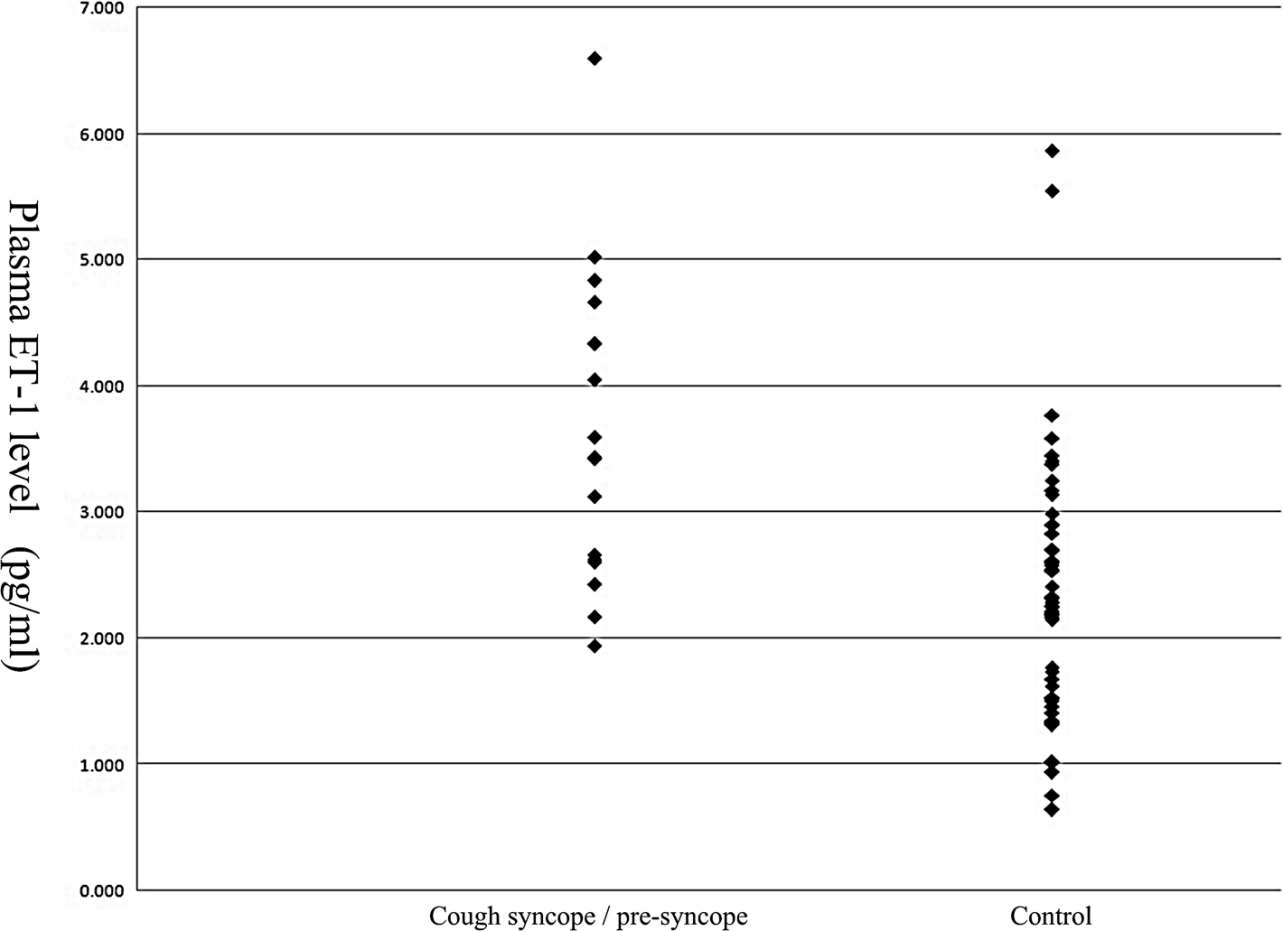
Plasma endothelin-1 levels in cough-syncope group (n = 17) and control group (n = 51).

The case group had higher frequencies of right-side, left-side, and both-side JVR, respectively (Table 1), and also higher plasma ET-1 levels (Figure 2) compared with control group. All JVRs detected in the case group occurred during the VM. Five patients of the cough syncope group and six of the cough pre-syncope group complained of dizziness during the VM. In the normal group, all right-side JVRs were detected during the VM, whereas in the left-side JVR, two (11.11%) were shown at baseline and 16 were detected during the VM.

### The presence of JVR and higher plasma ET-1 levels predicted the presence of cough syncope/pre-syncope

**Figure 3:**
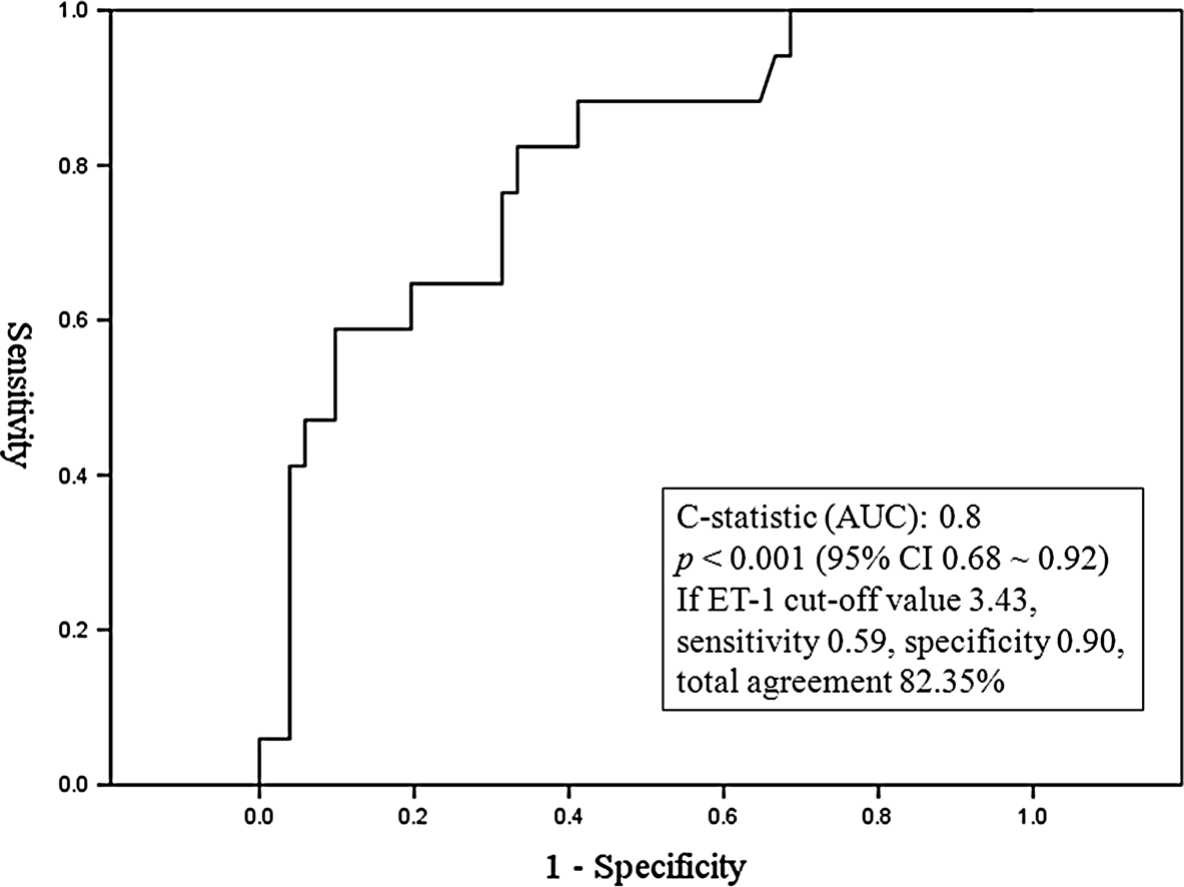
Receiver operator characteristic curve of the plasma endothelin-1 level in predicting cough syncope/pre-syncope patients.

ROC curve plotted for studying the utility of plasma ET-1 level in predicting cough syncope are shown in Figure 3. The area under the ROC curve (AUC) for ET-1 was 0.8 (*p* < 0.001, 95% confidence interval 0.68 ~ 0.92). The optimal cut-off point of ET-1 for predicting cough syncope was 3.43 pg/ml, with the sensitivity 0.59 and the specificity 0.90. Multivariate logistic analysis (Table 2) showed that the presence of both-side JVR and plasma ET-1 > 3.43 pg/ml were two independent factors associated with the presence of cough syncope/pre-syncope. Table 3 shows an interactive effect between both-side JVR and plasma levels of ET-1 on the occurrence of cough syncope/pre-syncope. There was a significant tendency that the presence of both-side JVR and plasma ET-1 > 3.43 pg/ml increased the frequency of cough syncope/pre-syncope (*p* < 0.001). There was less incidence of cough syncope/pre-syncope in subjects with the absence of both-side JVR and a plasma ET-1 ≦3.43 pg/ml, however, a significant increased trend of cough syncope/pre-syncope was seen in subjects with the presence of both-side JVR as well as a plasma ET-1 level > 3.43 pg/ml.

**Table 2:** Univariate and multivariate logistic analysis of factors associated with cough syncope/pre-syncope

	**Univariate**	***p *value**	**Multivariate**	***p *value**
HTN	1.26 (0.34-4.71)	0.729		
Diabetes mellitus	0.56 (0.06-5.30)	0.365		
Hyperlipidemia	0.73 (0.63-0.85)	0.307		
Smoking	0.79 (0.21-2.96)	0.729		
Obesity	0.62 (0.12-3.21)	0.518		
COPD	2.33 (0.59-9.24)	0.219		
Both-side JVR	7.68 (2.25-26.16)	0.001	10.77 (2.40-48.35)	0.0019
Plasma ET-1 > 3.43 pg/ml	10.35 (2.75-38.98)	0.0006	14.57 (2.95-71.59)	0.0010

Presented as OR (95% CI). Abbreviations: HTN = hypertension; COPD = chronic obstructive pulmonary disease; JVR = jugular venous reflux; ET-1 = endothelin-1.

**Table 3:** Frequencies of cough syncope/pre-syncope in four groups of subjects classified by the presence of both-side JVR and the levels of plasma ET-1

**Both-side JVR**	**Plasma ET-1, pg/ml**	**Cough syncope/pre-syncope**		***p*value**
		**-**	**+**	
No	≦ 3.43	38 (92.7%)	3 (7.3%)	<0.001
Yes	≦ 3.43	8 (61.5%)	5 (38.5%)	
No	> 3.43	5 (55.6%)	4 (44.4%)	
Yes	> 3.43	0	5 (100%)	

Presented as numbers (%). Abbreviations: JVR = jugular venous reflux; ET-1 = endothelin-1.

### No difference of ABP changes during VM between patients with cough syncope and normal controls

Ten patients with cough syncope [69.44(16.13) years; 9 males] and thirty age/gender-matched control subjects [69.44(16.13) years; 27 males] received the beat-to-beat ABP measurement at baseline and during the VM. The baseline and each phase of mean ABP between these two groups were similar [case group versus control group, mmHg; baseline=74.65(12.40) versus 77.91(14.33), *p* = 0.525; phase I=90.40(14.91) versus 99.59(18.05), *p* = 0.155; phase IIa=65.07(16.95) versus 73.61(17.43), *p* = 0.185; phase IIb=78.77(19.17) versus 88.63(21.33), *p* = 0.203; phase III=54.27(19.39) versus 62.58(17.80), *p* = 0.218; phase IV=83.97(20.55) versus 91.47(15.45), *p* = 0.229]. There was also no difference in each phasic change in mean ABP between case group and normal group [case group versus control group, %; phase I=21.46(7.87) versus 28.80(17.73), *p* = 0.216; phase IIa=−13.25(16.58) versus −5.61(15.84), *p* = 0.199; phase IIb=5.56(23.52) versus 14.08(23.64), *p* = 0.329; phase III=−27.88(22.05) versus −19.31(20.93), *p* = 0.275; phase IV=11.76(17.49) versus 19.41(23.17), *p* = 0.346]. Three patients with cough syncope complained of dizziness during the VM.

## Discussion

This pilot study is the first to prove that JVR is associated with cough syncope. Cough-induced JVR retrogradely transmits venous hypertension into the cerebral venous system, increases cerebral venous pressure or intracranial pressure, decreases CPP, and might consequently reduce CBF during cough [13-17]. There are other known facts supporting the idea that increased cerebral venous or intracranial pressure might play a role in the pathophysiology of cough syncope [26,27]. One study measured IJV venous pressure during cough in patients with cough syncope and found equalized IJV venous pressure with ABP [26]. In this situation, the net pressure gradient between ABP and venous pressure, the CPP, would decrease and lead to reduced CBF. Another study using transcranial Doppler (TCD) showed a cessation of forward flow in the cerebral artery and diastolic flow reversal during cough-induced syncope [27]. This hemodynamic finding during cough suggests increased impedance in the downstream circulatory pathway, such as in conditions with elevated cerebral venous pressure.

Another novel finding is that higher plasma levels of ET-1 could predict the occurrence of cough syncope. The plasma ET-1 levels have been recognized as a biomarker for predicting vascular endothelial dysfunction [28]. Whether patients with cough syncope have impaired cerebral endothelial function that makes them susceptible to cerebral hypoperfusion is worthy of further study.

The prevalence of JVR shown in a large-population study is around 20-40% [18]. If JVR contributes to the pathophysiology, the question remains why only certain people with JVR have developed cough syncope. There might be different additional factors combining with JVR involved in the pathophysiology of different diseases which are found associated with JVR. Our previous study has demonstrated an interactive effect between JVR and aging on the severity of age-related white matter changes [22]. We suggested that JVR adding age-related cerebral vascular abnormalities precipitated cerebral hypoperfusion in elderly people. In the present study, we have also found that, besides JVR, elevated plasma ET-1 levels might play an additional role in the pathophysiology of cough syncope. This could explain the mismatch between the incidence of cough syncope and VM-induced JVR.

Previous studies showed a fall in systemic ABP during or at the end of coughing, or during the VM in patients with cough syncope, and presumed cough-induced hypotension might play a role in the mechanism [23,24,29,30]. In our study, however, there was no significant difference in mean ABP changes during and at the end of the VM between case and control groups. It is possible that, compared with a virtual cough, the VM had a relatively insufficient intrathoracic elevation and resulted in a non-significant ABP decrement during the VM in the case group. Because of discrepant findings between present and previously reported cough syncope results, [23,24,29,30] we hypothesized that patients with cough syncope might have a higher central venous or intrathoracic pressure at baseline or/and during VM and VM-like activities (e.g. cough), and may induce vasovagal responses via baroreceptor stimulus during cough more easily than people without cough syncope. This could explain why people with cough syncope have both cough-induced hypotension and higher frequency of VM-induced JVR.

There are limitations in our pilot study. We enrolled relatively small sample size (n = 17) and used post-hoc data analysis approach. While simulating the pathophysiology of cough syncope, we evaluated JVR in subjects with supine position instead of upright position, and used VM instead of a heavy cough. Additionally, CBF changes were not analyzed in the present study. Therefore, we could not demonstrate the relationship between JVR/plasma ET-1 levels and CBF changes in our subjects with cough syncope. However, our previous study using transcranial Doppler did reveal an impact of JVR on CBF [15].

## Conclusions

JVR and higher plasma levels of ET-1 are associated with cough syncope/pre-syncope. Although sample size of this study was small, we showed a synergistic effect between JVR and plasma ET-1 levels on the occurrence of cough syncope/pre-syncope. Future studies should confirm our pilot findings.

## Abbreviations

ABP: Arterial blood pressure; CBF: Cerebral blood flow; CI: Confidence interval; CPP: Cerebral perfusion pressure; CSF: Cerebrospinal fluid; COPD: Chronic obstructive pulmonary disease; EEG: Eletroencephalography; EKG: Echocardiography; ET-1: Endothelin-1; GOLD: Global Initiative for Chronic Obstructive Lung Disease guideline; HTN: Hypertension; IJV: Internal jugular venous; JVR: Jugular venous reflux; NO: Nitric oxide; OR: Odds ratio; VM: Valsalva maneuver.

## Competing interests

The authors declare that they have no competing interests.

## Authors’ contributions

All authors made intellectual contributions to the study and approved the final manuscript. CPC, HYH, and KYY designed the study and performed the experiments. CPC carried out the neck color-coded duplex ultrasonography. KYY carried out the ET-1 assays. CPC, CYC, RZ, WCC, WYS, YCL, HHH, HYH, and KYY participated in the analysis and interpretation of data. CPC, HHH, HYH and KYY drafted the manuscript and are guarantors of the paper.

## Authors’ information

Dr. Hu is editor-in-chief of *Acta neurologica Taiwanica,* Professor of Neurology, National Yang-Ming University, and Chief of section of Neurovascular Diseases, Neurological Institute, Taipei Veterans General Hospital. Dr. Yang is an advisory editor of *Thoracic Medicine Taiwan* and Associate professor of Pulmonary Medicine, Institute of Clinical Medicine, School of Medicine, National Yang-Ming University.

## Pre-publication history

The pre-publication history for this paper can be accessed here:

http://www.biomedcentral.com/1471-2377/13/9/prepub
